# Overnight variations in cortisol, interleukin 6, tumour necrosis factor α and other cytokines in people with rheumatoid arthritis

**DOI:** 10.1136/ard.2007.086561

**Published:** 2008-03-28

**Authors:** M G Perry, J R Kirwan, D S Jessop, L P Hunt

**Affiliations:** 1 Academic Rheumatology Unit, University of Bristol, Bristol Royal Infirmary, Bristol, UK; 2 Henry Wellcome Laboratories for Integrated Neuroscience and Endocrinology, University of Bristol, Bristol; 3 Department of Clinical Sciences at South Bristol, University of Bristol, Bristol, UK

## Abstract

**Objective:**

To investigate overnight variations in absolute values and patterns of cytokines including interleukin 6 (IL6) and tumour necrosis factor α (TNFα) in rheumatoid arthritis (RA), and to relate any changes to those occurring in blood cortisol.

**Methods:**

A total of 16 people (8 female) with active RA and who had received no recent glucocorticoids were admitted overnight. Blood samples were obtained at 13 time points between 21.00 and 10.00.

**Results:**

The geometric mean IL6 concentration rose significantly from 35 pg/ml at 22:00 to 64 pg/ml at 07:15 (repeated measures analysis of variance (ANOVA), p<0.001). The geometric mean cortisol concentration rose significantly overnight from 57 ng/ml at 01:00 to 229 ng/ml at 07:15 (repeated measures ANOVA, p<0.001). Neither TNFα nor the other cytokines measured changed significantly. Using cubic regression modelling IL6 began to rise before cortisol (range 0.01 to 4.83 h) in eight participants and after cortisol (range 1.11 to 5.14 h) in three participants. In a random coefficient model including data from all participants, the estimated mean IL6 value began to rise 3.05 h before the estimated mean cortisol value, with the IL6 peak occurring 0.70 h before the cortisol peak.

**Conclusion:**

The mean IL6 and cortisol concentrations showed a significant overnight variation. Neither TNFα nor the other cytokines measured changed significantly. In a random coefficient model IL6 began to rise approximately 3 h, and reached a peak about 40 min, before cortisol. These studies confirm that there are abnormalities in plasma cortisol and IL6 concentrations and dynamics. The data also link the overnight rise in IL6 to the circadian variation in symptoms.

Patients with rheumatoid arthritis (RA) report joint pain, swelling and stiffness on waking, which improve as the morning progresses. This “early morning stiffness” is considered a basic feature pointing to inflammatory polyarthritis. Circadian variation in symptoms and signs was first recorded 25 years ago^1^ and animal models of inflammatory arthritis show similar patterns.^2^ ^3^ In RA synovitis is driven in part by excessive production of cytokines such as interleukin (IL)6 and tumour necrosis factor α (TNFα).^4^ The hypothalamic–pituitary–adrenal (HPA) axis and its associated circadian variations in cortisol are also implicated.^5^ Since the anti inflammatory properties of cortisol were first described,^6^ studies have failed to pinpoint a precise defect in HPA control.^5^ ^7^ Sequential 24-h blood samples suggest the HPA axis is unable to mount an appropriately enhanced glucocorticoid response to combat joint inflammation.^1^ ^8^^–^11

Serum concentrations of the proinflammatory cytokines IL1β, IL6 and TNFα are raised in RA, correlate with disease activity, and fall in response to treatment.^12^ ^13^ Furthermore, recombinant IL6 dramatically stimulates the HPA axis.^14^ Thus, there is the potential for a link between the inflammatory cytokines and circadian cortisol control. Variation in IL6 in RA was reported when measured every 3 h between 07:30–22:30^15^ and when measured over 24 h in five newly-diagnosed patients a peak occurred at 07:00.^10^ In two studies of low dose glucocorticoid (prednisolone <7.5 mg equivalent) given at 02:00 compared with 07:30, IL6 and symptoms were both improved at 08.00.^16^ ^17^ Thus, it is possible that variation in IL6 is related to variation in symptoms.

For TNFα and IL1β, one^11^ of two studies reporting overnight variations had a large difference between concentrations at the start and at the end of the 24-h measurements (that should be similar in circadian variation) and so may not have been true biological variation. In the other,^18^ the variation in TNFα was not statistically significant. A subsequent review^22^ recalculated the values as percentages of the 24-h mean, which overemphasises relatively small changes. Therefore, in order to clarify the true nature and extent of nocturnal variations in cytokines and their inter-relationship, we measured several cytokines simultaneously at frequent intervals during the night to determine directly the absolute changes in cytokine levels, their relative importance and their relationship to blood cortisol. Our analysis focuses principally on mathematical modelling of these patterns.

## PATIENTS AND METHODS

### Patients

The study was approved by the United Bristol Healthcare NHS Trust (UBHT) Research Ethics Committee and complied with the Helsinki Declaration. Following written informed consent 16 patients with RA^19^ and active disease (⩾3 swollen and ⩾3 tender joints) were included who had received no glucocorticoids by any route in the previous 11 weeks (except patient 9: intra-articular triamcinolone hexacetonide 40 mg 36 days earlier) and no biological therapy at any time. All female patients were postmenopausal. Three healthy control volunteers served to ensure that blood sample collection did not unduly disturb normal circadian variation.

### Procedure

Participants attended at 19:00 and generally remained on bed rest. An intravenous cannula, usually inserted in the antecubital fossa using local anaesthetic, was flushed immediately with 10 ml 0.9% saline (repeated before and after each blood sample) and left for at least 1 h. Samples were taken at 21:00, 22:00, 23:00, 01:00, 03:00, 04:00, 05:00, 05:45, 06:30, 07:15, 08:00, 09:00 and 10:00, and the first 3 ml of each discarded to avoid dilution. Low-level lighting was used when participants wanted to sleep.

### Clinical assessments

Assessments, performed between 08:00 and 10:00, included tender and swollen 28-joint counts, morning stiffness (in min), pain (on a 10 cm visual analogue scale: no pain to severe pain), patient and clinician’s opinions of RA activity (on 10 cm visual analogue scales: no disease activity to severe disease activity) and the Hospital Anxiety and Depression Scale (HADS).^20^ Height and weight were measured and the body mass index (BMI) and 28-joint Disease Activity Score (DAS28) calculated.

### Laboratory measurements

Plasma was separated and stored at –80°C within 1 h of collection. The routine hospital laboratory measured C-reactive protein (CRP), plasma viscosity, haemoglobin and rheumatoid factor. Total plasma cortisol was measured by in-house radioimmunoassay using antisera raised in rabbits to cortisol-3-(*O*-carboxymethyl) oxime (Bioclinical Services, Cardiff, UK). Tracer was cortisol-3-(*O*-carboxymethyl) oximino-(2-[^125^I]iodohistamine (Amersham Pharmacia Biotech, Little Chalfont, UK). Intra- and interassay coefficients of variation for cortisol were 5 and 8–12% respectively. Plasma cytokine analyses for TNFα, IL1β, IL6 and IL8 were performed on a Luminex 100 (Luminex, Austin, Texas, USA) using ACS software (limit of detection 2.4 pg/ml), and for IL4, IL5, IL10, IL13 and IFNγ on a Bio-Rad Bio-Plex system (limit of detection 0.01 pg/ml). Values below the limit of detection were set at the limit. The intra assay variability was 2.9% for TNFα, 4.1% for IL6 and IL8 and 5.8% for IL1β. All plasma assays for cortisol and cytokines were performed within 8 months.

### Statistical analysis

Cortisol and cytokine concentrations were logarithmically transformed (base 10) to remove skewness and summarised as geometric means. Repeated measures analysis of variance (ANOVA) was used to determine whether there were changes in mean blood concentrations for each of the constituents over time in the patient group as a whole (“proc MIXED”, SAS V. 8.2: SAS Institute, Cary, North Carolina, USA,) using either antedependence or spatial power correlation structures. Individual patterns of patient cortsol and IL6 were explored using a cubic regression model (y = at^3^+bt^2^+ct+d, where t = time in h) and the values and timings of troughs (minima) and peaks (maxima) estimated from the model coefficients. We arbitrarily discounted extrapolation more than 3 h outside the period of observation. In a separate analysis of the patients as a group, a random coefficient cubic regression model was fitted to all the patient data using “proc MIXED”. Each of the coefficients was assumed to vary across patients and their means variances and covariances estimated. In an exploratory analysis, cortisol and IL6 concentrations in the eight patients with the highest scores for morning stiffness (and separately, DAS28) were compared to those in the eight with the lowest scores using the random coefficient cubic regression model to compare the values and timing of the troughs and peaks in the model, so as to identify any change in pattern related to these two variables.

## RESULTS

There were 8 men and 8 women (table 1), mean age 59 years (range 38–75) and mean disease duration 10 years (range 0.2–30); 15 were taking non-steroidal anti-inflammatory drugs and 12 taking disease-modifying antirheumatic drugs (methotrexate 6, sulphasalazine 2, methotrexate and sulphasalazine combination 2, leflunamide 1, penicillamine 1). The mean BMI was 26 (21–38). Three participants were obese with a BMI 30 (patients 5, 12 and 15) but had cortisol profiles that were similar to the other non-obese participants (BMI<30). The mean HADS anxiety score was 6.6 (1–12) and the HADS depression score was 4.9 (1–13). One participant (patient 3) had a HADS depression score of 13, indicating possible depression. This participant and two others (patients 4 and 5) had HADS anxiety scores of 12, 12 and 11, respectively, indicating possible anxiety. Patients had a mean CRP of 53 mg/litre (10–159), plasma viscosity 1.76 mPa (1.46–2.17), haemoglobin 12.2 g/dl (9.6–17.7) and DAS28 5.05 (4.1–6.3); 11 (69%) were rheumatoid factor positive and 12 (75%) had radiological evidence of erosions.

**Table 1:** Summary of clinical characteristics of patients*

Characteristic	Mean (SD)
Male/female	8/8
Age (years)	59.1 (10.1)
Disease duration (years)	10.2 (9.2)
Erosive disease (%)	80
Haemoglobin (mg/dl)	12.2 (1.9)
Plasma viscosity (mPa)	1.8 (0.2)
C-reactive protein (mg/litre)	47.3 (46.3)
Rheumatoid factor positive (%)	70
Weight (kg)	72.3 (14.7)
Height (m)	1.7 (0.1)
BMI (kg/m^2^)	26.2 (4.6)
Tender joint count	13.1 (6.6)
Swollen joint count	9.1 (4.9)
Early morning stiffness (min)	81.6 (82.8)
Patient global opinion (cm)	5.1 (2.2)
Pain (cm)	4.8 (2.5)
Clinician global opinion (cm)	5.9 (2.4)
DAS28	5.1 (0.8)
HADS (A)	6.6 (3.4)
HADS (D)	4.9 (3)

*Full details of all patients are available in the supplementary material.

BMI, body mass index; DAS28, 28-joint Disease Activity Score; HADS, Hospital Anxiety and Depression Scale.

### Laboratory results

**Figure 1:**
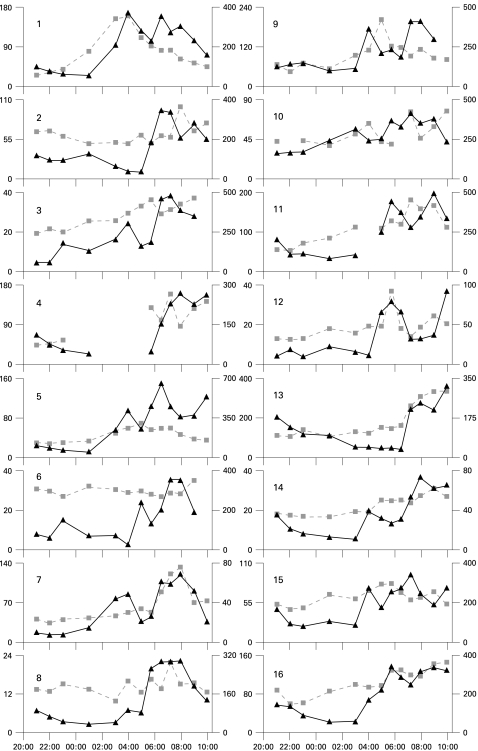
Charts of individual data for all patients showing interleukin (IL)6 (squares, left axis, pg/ml) and cortisol (triangles, right axis, ng/ml).

The pattern of plasma cortisol variation in healthy volunteers was similar to that previously reported: at 22:00 geometric mean (95% CI) cortisol was 8.6 ng/ml (3.3 to 22.3) and at 05:45 the peak was 56.9 ng/ml (21.5 to 150.6). Individual cortisol concentrations for patients with RA are shown in fig 1 (provided in detail in the supplementary material). The minimum geometric mean was 56.9 ng/ml (37.2 to 87.1) at 01:00 and the maximum was 228.7 ng/ml (146.7 to 356.7) at 07:15 (repeated measures ANOVA p<0.001).

In healthy volunteers IL6 remained low for most of the night but showed a small rise in the early hours of the morning. The minimum geometric mean (95% CI) was 15.3 pg/ml (8.0 to 29.2) at 21:00 and the maximum was 19.7 pg/litre (7.4 to 52.0) at 08:00. Other cytokines showed no consistent pattern. Individual IL6 concentrations for patients with RA are shown in fig 1 (summarised in the supplementary material). The geometric mean changed significantly with time (repeated measures ANOVA p<0.001), with a minimum of 34.5 pg/ml (24.5 to 48.2) at 22:00 and a maximum of 64.4 pg/ml (41.2 to 100.6) at 07:15. The geometric means of TNFα did not change significantly with time (p = 0.202; see supplementary material). Although other cytokines were difficult to analyse because of values below the limit of detection, exploratory analyses failed to detect significant overnight changes in IL1β, IL4, IL10 and IL13 (see supplementary material). Too few IL5, IL8 and IFN results were above the limit of detection for further assessment.

### Statistical modelling

In one participant (patient 3) no minima or maxima could be found for cortisol and in two (patients 12 and 13) the peak would have fallen more than 3 h outside the period of observation. The calculated values and times of the peaks and troughs for the remaining participants are shown in the supplementary material. As IL6 was the only cytokine showing statistically significant variations over time, regression modelling was applied to IL6 but not to the other cytokines. For IL6 there were two participants where no minima or maxima could be found. There were three participants where the trough, and two where the peak, would have fallen more than 3 h outside the period of observation. Analysis of the remaining peaks and troughs showed that the IL6 began to rise before cortisol (range 0.01–4.83 h) in eight patients and after cortisol (range 1.11–5.14 h) for three patients.

**Figure 2:**
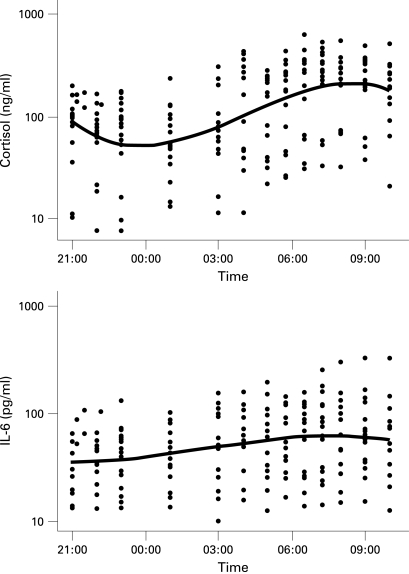
Model best fit lines for cortisol and interleukin (IL)6 with log transformed values shown for each patient as dots.

**Figure 3:**
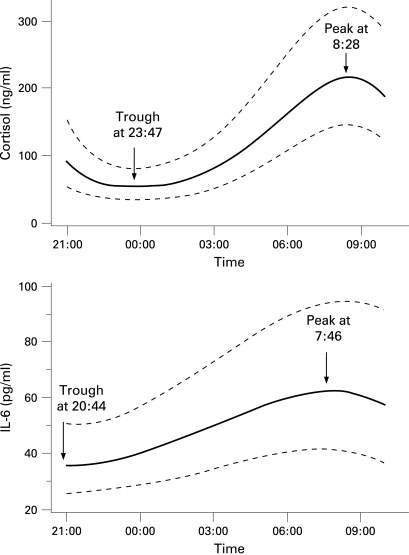
Model best fit lines with 95% confidence intervals for cortisol and interleukin (IL)6 with absolute values and minima and maxima indicated.

The definitive random coefficient models including all (log transformed) data from all patients for cortisol and for IL6 and are shown in fig 2 and in the supplementary material. The rise in mean IL6 concentration began 183 min before cortisol, with peak IL6 occurring 42 min before cortisol, as illustrated using the back-transformed model results in fig 3.

### Clinical assessments

These showed correlation between tender and swollen joint counts (r = 0.76, p<0.01) and between each of these and the DAS28 (r = 0.88, p<0.01; r = 0.88, p<0.01). DAS28 also correlated with clinician global (r = 0.54, p<0.05), but not morning stiffness (r = 0.32, not significant (NS)) or patient global (r = 0.29, NS). Morning stiffness correlated with patient global (r = 0.63, p<0.01) and pain (r = 0.75, p<0.01). There was no difference between high and low morning stiffness patients in the models of overnight variation in cortisol or IL6. For cortisol there was a significant difference (p = 0.048) between patients with high and low DAS28 scores. Patients with high DAS28 scores had a cortisol trough of 48 ng/ml at 23:10 compared to those with lower DAS28 scores of 55 ng/ml at 00:30 (time difference 80 min) and peaks of 225 ng/ml at 07:38 and 247 ng/ml at 10:06 (time difference 148 min).

## DISCUSSION

We measured 9 cytokines at frequent intervals during the night in 16 patients with active RA, extending previous but less extensive observations,^10^ ^15^ ^18^ substantially increasing the number of patients for whom night-time cortisol and cytokine data are available, and for the first time allowing a direct comparison of the absolute changes in concentrations of the different cytokines and their relative timing and importance. The variation in cortisol was similar to that previously reported,^1^ ^8^^–^11 21 with the average trough at 23:47 and peak at 08:28. While concentrations of TNFα, IL1β, IL4, IL6, IL8 and IL10 were raised compared to normal volunteers (in agreement with the literature) the only cytokine to show significant overnight variation was IL6. In the majority of patients, plasma IL6 rose before cortisol, although the reverse was found in a few patients, possibly reflecting the timing of particular samples or a poorly fitting statistical model. Taking all the patients together, IL6 began to rise approximately 3 h before plasma cortisol, and reached a peak about 40 min before cortisol. The results in our normal volunteers showed concentrations similar to those reported in the literature,^10^ ^22^ ^23^ suggesting that the overnight procedures we used were not substantially disruptive to established circadian variations.

In this study, neither TNFα nor IL1β, nor any of the other measured cytokines, showed significant overnight variation in absolute values. This contrasts with two earlier reports^11^ ^18^ that we believe were inconclusive (see above). In a review of the cytokine data available at the time,^24^ the conclusion that TNFα showed circadian variation in patients with RA was dependant on these two studies and on recalculation of TNFα concentrations as percentages of the 24-h mean. This process ignores absolute values and exaggerates small (and non-significant) changes. Expressing the data from our study in a similar manner appears to show marked TNFα variations, but in fact they are minor and statistically non-significant when measured in absolute terms. These difficulties in interpreting previous studies emphasises the need for frequent and simultaneous overnight sampling of all relevant cytokines, as reported here.

We chose cubic regression modelling for our principal analysis. This allowed for fitting to a trough and a peak, expected to occur overnight, and enabled us to estimate the times and values for the troughs and peaks for individual patients, and for the best fit model for all the patient data taken together. Circadian modelling includes a 24-h period and for these models additional harmonic terms would need to provide an appropriate fit, increasing the number of parameters estimated and demanding a greater range of data. Our measurements were available for only 13 h, which was sufficient for the cubic regression modelling, but would not support circadian modelling. While in future studies it may be advantageous to extend blood sampling to 24 h in order to check circadian models against previously published work, the possibility remains that concentrating on the times of interest may provide greater precision.

Some authors have suggested that an intravenous cannula may itself cause raised IL6 concentrations, possibly through “local” IL6 production.^25^^–^29 This literature suggests the blood IL6 level may be increased by up to 1 pg/ml/h but that TNFα and other cytokines are unaffected. We were able to take an extra sample from the opposite arm at the end of each study for three patients and three controls, and measure IL6 in the same assay as the sample from their cannula. We found the opposite arm sample to be 21% lower (on average), in accordance with this theory of local production. This difference would affect the final absolute values in our results, and deserves further investigation, but it would not affect the overall pattern of our results and their interpretation.

Studies of this type demand a large commitment, in particular from patients and healthy volunteers, so the number of subjects, while comparable to other papers in the field, is relatively small. The broad pattern of the results for IL6 and cortisol will require confirmation, but lack of significant changes in other cytokines was consistent across all the patients studied. It is possible that repeated blood sampling disturbed circadian variation. Many people with RA (including the participants in this study) describe poor sleep in their usual environment.^30^ As the study progressed we collected informal assessments suggesting disturbance was not great (data not shown) and we will include formal measurements in future studies. Some authors have suggested that age^22^ and sleep deprivation^23^ ^31^ alter the IL6 concentration. However, the reported changes are small and unlikely to have made a substantial difference to our measurements, which for IL6 were up to 20 times normal and for TNFα 40 times normal. Our study was not circadian because it was only overnight (13 h) and did not last 24 h. This period was chosen for practical reasons and because this was when the relevant changes in cortisol and IL6 were likely to occur. Now the variations in cortisol and IL6 are confirmed, future studies might best collect 24-h data.

Our clinical data showed that morning stiffness and DAS28 were poorly correlated and related to different patient characteristics. This raised the possibility that they represent different aspects of the disease and so we sought evidence for this from within our cytokine measurements. Small but significant difference in the timing of the overnight variations in cortisol between patients with higher and lower DAS28 scores also raises the possibility that patterns of HPA response are related to overall disease activity.

Of the many studies examining the HPA axis in RA,^5^ ^7^ only three include overnight data for relevant cytokines^10^ ^11^ ^18^ and, as discussed above, in two it is difficult to draw firm conclusions. In the third, Crofford *et al*^10^ studied five newly-diagnosed patients and showed an IL6 increase preceded adrenocorticotropic hormone (ACTH), which preceded cortisol, with approximately 1 h between each. Our data, in 16 patients with a wide range of disease duration, support and expand upon this finding. These studies confirm that there are abnormalities in plasma cortisol and IL6 concentrations and dynamics. They also link the overnight rise in IL6 to the circadian variation in symptoms. We and others have suggested that a low dose of glucocorticoid medication, delivered at 02:00 is more effective at reducing symptoms and plasma IL6 concentration (in a single 08:00 blood sample) than an equivalent glucocorticoid dose taken at 07:00.^16^ ^17^ If this were confirmed in future studies, it may have important consequences for the dose and timing of oral glucocorticoid treatment.
